# Prevalence of antibiotic-resistant *Acinetobacter* spp. on soil and crops collected from agricultural fields in South Korea

**DOI:** 10.1007/s10068-023-01496-7

**Published:** 2024-01-29

**Authors:** Su Min Son, Eunbyeol Ahn, Sojin Ahn, Seoae Cho, Sangryeol Ryu

**Affiliations:** 1 Department of Food and Animal Biotechnology, Department of Agricultural Biotechnology, Research Institute of Agriculture and Life Sciences, Seoul National University, Seoul, 08826 Republic of Korea; 2 Center for Food and Bioconvergence, Seoul National University, Seoul, 08826 Republic of Korea; 3 Interdisciplinary Program in Bioinformatics, Seoul National University, Seoul, 08826 Republic of Korea; 4 eGnome Inc., Seoul, 05836 Republic of Korea

**Keywords:** *Acinetobacter*, Agriculture, Antibiotic resistance, Biofilm, Motility

## Abstract

The emergence of antibiotic resistance in *Acinetobacter* spp. is a rising public health concern worldwide. The objective of this study was to investigate the prevalence of antibiotic-resistance genes and the virulence of *Acinetobacter* spp. isolated from soil and crops obtained from agricultural fields in South Korea. Eight *Acinetobacter* spp. isolates carried various antibiotic resistance genes, such as *emrAB* (100%), *ca*t/*craA* (100%), and *aadA* gene (87.5%). Minimum inhibitory concentration (MIC) analysis revealed that strains harboring antibiotic resistance genes exhibited high resistance to the respective antibiotics, such as colistin, chloramphenicol, and streptomycin. Interestingly, most of these isolates had high capability of biofilm formation and swarming motility, along with faster growth rates. Taken together, our study demonstrated that antibiotic-resistant *Acinetobacter* isolated from agricultural settings in South Korea not only frequently carries antibiotic resistance genes but also has virulence-related traits.

**Supplementary Information:**

The online version contains supplementary material available at 10.1007/s10068-023-01496-7.

## Introduction

*Acinetobacter* spp. are Gram-negative, aerobic, opportunistic bacteria which are widely distributed in hospital environments or hospitalized patients, and also in soil and water (Cisneros and Rodríguez-Baño, 2002). *Acinetobacter* spp. can survive on either dry or moist surfaces for long periods and grow at a range of different temperatures and pH values. *Acinetobacter* spp. are also known to form biofilms, known as the most effective virulence factor, which further compromises the use of antibiotics in treating infections. *Acinetobacter* spp., especially *A. baumannii,* have been numerously reported for increasing involvement in outbreaks in clinical settings. Furthermore, the emergence of multidrug-resistant (MDR) *Acinetobacter* has led to a rise in public health concerns (Carvalheira et al., 2021). According to the World Health Organization (WHO), one of the most emphasized antibiotic-resistant “priority pathogens” for which new antibiotics are urgently needed is *A. baumannii* (Tacconelli et al., 2018)*.* Antibiotic resistance genes are easily transferred in *Acinetobacter* especially by conjugation, leading to the increasing emergence of MDR *Acinetobacter* (Leungtongkam et al., 2018).

Recent studies report that *Acinetobacter* spp. can be introduced into the hospital environment through kitchens or by food (Campos et al., 2019). Several studies have also reported that MDR *Acinetobacter* spp. is found in agricultural products such as lettuce and fruits (Carvalheira et al., 2017). MDR *Acinetobacter* spp. poses a major threat to hospitals causing severe nosocomial infections that are related to high mortality rates. However, few studies have reported the prevalence of MDR *Acinetobacter* spp. in agricultural environments in South Korea (Ababneh et al., 2022; Kim et al., 2016). The purpose of this study was to investigate the prevalence of antimicrobial resistance of *Acinetobacter* spp. among soil and agricultural products from agricultural fields in South Korea and analyze their virulence-related traits.

## Materials and methods

### Isolation of *Acinetobacter* spp. from agricultural environments in South Korea

Soil and crops from agricultural environments were collected randomly from different local fields located in three Korean provinces, Seoul, Jeju, and Chungnam, using standard procedures in September 2022. Collected samples were enriched with Luria–Bertani (LB) broth (Difco, Detroit, MI, USA) at 37°C overnight and plated onto the selective agar medium of *Acinetobacter*, CHROMagar™ Acinetobacter (CHROMagar, Paris, France). The plates were incubated at 37°C overnight. Colonies on plates were picked and confirmed with PCR using specific primers for *Acinetobacter* and 16S rRNA sequencing (see supplementary data Table S1). Isolated *Acinetobacter* spp. were stored in glycerol stock at − 80°C. The *Acinetobacter* spp. isolates were grown on LB media at 37°C.

### Whole genome sequencing and bioinformatics analysis

Genomic DNA (gDNA) was extracted using PureHelix Genomic DNA Prep Kit (Solution Type)-Bacteria (Nanohelix, Daejon, Korea). Then, gDNA was quantified and qualified by gel electrophoresis 260/230 nm and 260/280 nm absorbance ratio and Quant-iT™ PicoGreen^R^ dsDNA Assay Kit (Invitrogen). Following the manufacturer’s guidelines, a Nanopore MinION long-read sequencing library was prepared using a ligation sequencing-native barcoding kit (SQK-NBD114.24; Oxford Nanopore Technologies, UK) and sequenced on a FLO-MIN114 (R10.4.1) flow cell with a MinION Mk1B device and MinKNOW software (22.10.7). De novo assembly were conducted using Flye version 2.9.1 (Kolmogorov et al., 2019). Pangenome analysis was performed using Roary Pangenome Pipeline (Page et al., 2015). Roary was used (with a 95% BLASTp percentage identity cut-off) to cluster the genes encoding complete protein sequences into core (hard core and soft core) and accessory (shell and cloud) genomes.

### Growth curve

Overnight cultures were diluted 1:100 in fresh LB broth in a 24-well plate. Growth was monitored every 15 min for 12 h by measuring optical density at 600 nm (OD_600_) using the SpectraMax i3 Plus Microplate Spectrophotometer (Molecular Devices, USA). Experiments were conducted in triplicates.

### Antimicrobial susceptibility testing

Antimicrobial susceptibility was determined using a broth dilution method according to the Clinical and Laboratory Standards Institute (CLSI) guideline (CLSI, 2020). The minimum inhibitory concentrations (MICs) of colistin (Sigma), chloramphenicol (Duchefa), streptomycin (Sigma), gentamycin (Sigma), piperacillin (Duchefa), cefotaxime (Sigma), tetracycline (Sigma), ciprofloxacin (Sigma), doripenem (Sigma), imipenem (Sigma), meropenem (Sigma, Saint Louis, MO, USA) were determined using *E. coli* ATCC 25922 (American Type Culture Collection; ATCC, Manassas, VA, USA) as a quality control strain (CLSI, 2020). The antibiotic resistance level of chloramphenicol and streptomycin was determined based on previous studies (Wei and Yang, 2017; Yang et al., 2019).

### Biofilm formation assay

The biofilm formation capacity of each strain was estimated using the crystal violet staining method described previously (Cha et al., 2019). Briefly, strains were cultured in LB broth overnight and was diluted to reach OD_600_ = 0.1 with fresh LB broth in 96-well plates. After incubation at 37°C for 24 h in static conditions, the plates were washed three times with PBS (Dulbecco’s Phosphate-Buffered Saline, GenDEPOT), and each well was stained with 200 μl of 0.1% crystal violet (CV) for 20 min. The plates were again washed three times to remove excess stain, and solubilized with 200 μl of 33% acetic acid for 20 min. The OD_570_ was then measured using the microplate spectrophotometer, to calculate the biofilm formation capacity of the isolates. The extent of biofilm formation of each isolate was compared to the reference strain, *A. baumannii* CCARM 12001, which is known as a multidrug-resistant strain. *A. baumannii* CCARM 12001 was obtained from the Culture Collection of Antimicrobial Resistant Microbes, Korea. Un-inoculated LB broth was used as a negative control. All experiments were carried out in triplicates.

### Swarming motility assay

Freshly grown cultures were stabbed to enable spread of bacteria on the surface of the medium (0.4% semisolid) as described previously (Choi et al., 2013). Modified LB broth (tryptone—10 g/l; NaCl—5 g/l; yeast extract—5 g/l; micro-agar—4 g/l) was used for the motility assay (Vijayakumar et al., 2016). Plates were prepared on the same day as the inoculation. A 2 μl aliquot of an overnight culture diluted to an OD_600_ of 1 was spotted in the middle of the plate and allowed to dry at room temperature. Then, plates were incubated at 37°C for 24 h. Experiments were performed at least three times.

## Results and discussion

### Isolation and identification of *Acinetobacter* isolates

**Fig. 1:**
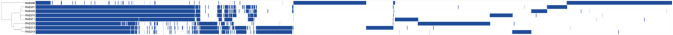
Phylogenetic tree and pangenome analysis of *Acinetobacter* isolates. Each row corresponds to a branch on the tree. Blue indicates gene presence and white indicates gene absence. The phylogeny reflects the clustering of complete protein sequence encoding genes into core and accessory genomes

Eight strains of *Acinetobacter* spp. were isolated from soil and crops from agricultural fields in South Korea. Various *Acinetobacter* spp. was identified including *A. seifertii, A. vivianii, A. pittii,* and *A. baumannii* isolates (Table 1). *A. seifertii* and *A. pittii* are close relatives of *A. baumannii* isolates, the control of which is critical in clinical settings (Towner, 2009). The phylogenetic tree of the 8 isolated strains was constructed, showing that the isolates could be divided into 2 big clades, where RAES06 is distinctly separated from the rest of the 7 isolates (Fig. 1).

**Table 1:** Isolates of *Acinetobacter* in this study

Name	Strain	Source	Location	Accession No.
RAES01	*A. pittii*	Soil	Seoul, Korea	CP133067
RAES03	*A. seifertii*	Coltsfoot	Jeju, Korea	CP133074
RAES06	*A. vivianii*	Soil	Jeju, Korea	CP133075
RAES10	*A. pittii*	Pumpkin leaves	Chungnam, Korea	CP133068
RAES11	*A. pittii*	Peas	Chungnam, Korea	CP133069
RAES13	*A. baumannii*	Soil	Chungnam, Korea	CP133070
RAES19	*A. baumannii*	Soil	Chungnam, Korea	CP133072
RAES20	*A. pittii*	Soil	Chungnam, Korea	CP133073

### Antimicrobial resistance profiles and pangenome analysis of *Acinetobacter* isolates

Antibiotic susceptibility results showed that *Acinetobacter* isolates were highly resistant to colistin (8/8, 100%), chloramphenicol (7/8, 87.5%), and streptomycin (4/8, 50%), whereas all isolates were susceptible to gentamycin, piperacillin, cefotaxime, tetracycline, ciprofloxacin, and carbapenems (Table 2). Since previous studies have reported that *mcr-1* gene was detected in colistin-resistant *Acinetobacter*, we tested the presence of *mcr-1* gene in the isolates (Hameed et al., 2019; Kalová et al., 2021). The *mcr-1* gene was not detected in all isolates based on PCR results (data not shown). However, the *emrA* or *emrB* gene, which encodes efflux pumps associated with colistin resistance in *Acinetobacter,* was present in all isolates (see supplementary data Table S2) (Boinett et al., 2019; Lin et al., 2017). Based on the pangenome analysis, we discovered poor correlation between the *aadA* gene and streptomycin resistance in isolates (Fig. 1 and Table S2). The *aadA* gene, which encodes 3′′-adenylyltransferase, causes resistance to streptomycin and spectinomycin (Svab et al., 1990). All isolates, except RAES03, encoded the *aadA* gene, but only 50% of the isolates showed high resistance to streptomycin (see supplementary data Table S2). This result may be explained by poor expression or silencing of the *aadA* gene (Schmidt et al., 2018). All isolates also encoded the *cat* and the *craA* gene, which are both related to chloramphenicol resistance, and all isolates except RAES06 showed high resistance to chloramphenicol (see supplementary data Table S2) (Devaud et al., 1982; Roca et al., 2009). It is notable that *Acinetobacter* isolates from agricultural fields are mostly resistant to colistin, chloramphenicol, and especially to streptomycin, given the prevalent and abundant use of antibiotics as pesticides in South Korea (Lee et al., 2021; Sundin and Wang, 2018).

**Table 2:** Minimum inhibitory concentrations (MICs) of different antibiotics against *Acinetobacter* isolates

Strain	MIC (μg/mL)										
	COL	CHL	STR	GEN	PIP	CTX	TET	CIP	DOR	IPM	MEM
RAES01	**8**	**16**	**16**	1	16	8	0.5	< 0.125	0.125	0.25	1
RAES03	**8**	**8**	4	2	16	4	0.5	0.25	0.125	0.25	1
RAES06	**4**	4	2	1	16	4	0.5	< 0.125	0.5	0.5	2
RAES10	**8**	**32**	8	1	16	8	0.5	0.25	0.125	0.125	1
RAES11	**8**	**32**	8	1	16	8	0.5	0.25	0.25	0.25	1
RAES13	**8**	**32**	**16**	2	4	4	1	0.25	0.125	0.25	1
RAES19	**8**	**32**	**16**	2	16	8	0.5	< 0.125	0.125	0.25	1
RAES20	**8**	**32**	**16**	2	8	8	1	< 0.125	0.25	0.5	1
Total (%)	8/8 (100%)	7/8 (87.5%)	4/8 (50%)	0/8 (0%)	0/8 (0%)	0/8 (0%)	0/8 (0%)	0/8 (0%)	0/8 (0%)	0/8 (0%)	0/8 (0%)

*COL* colistin, *CHL* chloramphenicol, *STR* streptomycin, *GEN* gentamicin, *PIP* piperacillin, *CTX* cefotaxime, *TET* tetracycline, *CIP* ciprofloxacin, *DOR* doripenem, *IPM* imipenem, *MEM* meropenem

MICs considered resistant are highlighted in bold

### Growth rates of *Acinetobacter* isolates

**Fig. 2:**
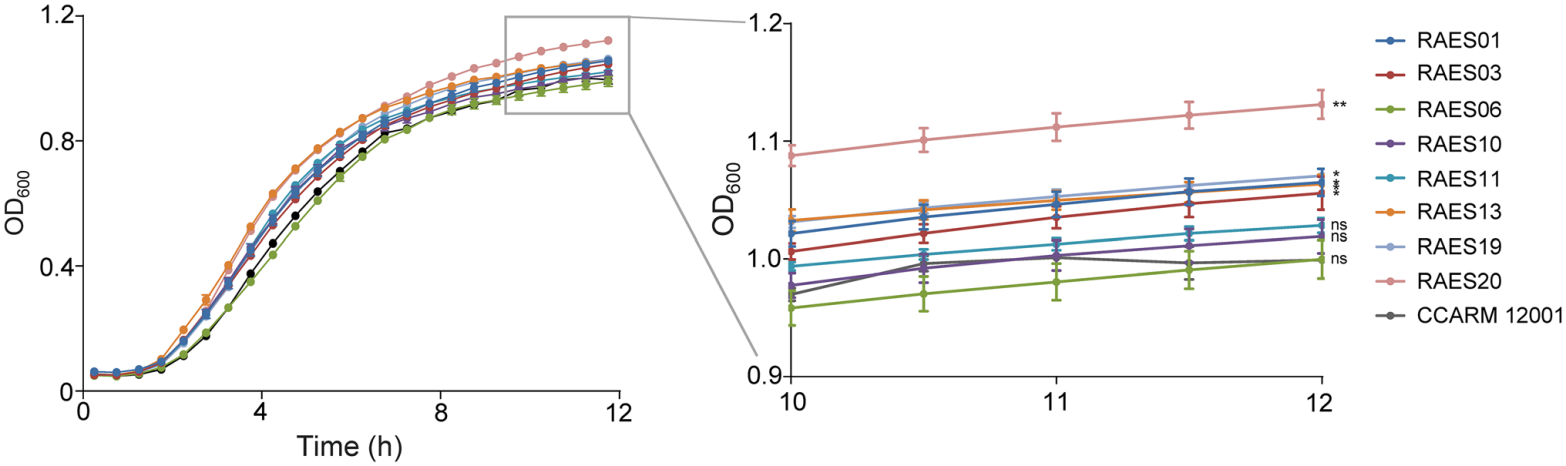
Growth curve of *Acinetobacter* isolates. Growth was monitored every 30 min for 12 h. The inset shows a magnified view at the end of the growth curve. Experiments were repeated three times and the data represent mean ± standard deviation. Results were analyzed based on CCARM 12001 results by the Student’s *t*-test. *P < 0.05; **P < 0.01; ns, not significant

The acquisition of antibiotic resistance is known to impair bacterial fitness resulting in a growth defect (Andersson and Hughes, 2010; Song et al., 2014). To estimate the growth rates of *Acinetobacter* spp. isolates, we measured bacterial growth with the MDR *A. baumannii* CCARM 12001 as a control strain. Most isolates exhibited significantly higher growth rates compared to the control strain (Fig. 2). Notably, RAES13, RAES19 and RAES20 showed a more rapid exponential growth phase compared to the other strains, with RAES06 exhibiting the slowest growth (Fig. 2). Interestingly, the fast-growing isolates showed the highest antibiotic resistance (27.3%) among the eight isolates, whereas RAES06 was the most susceptible (9.0%) to antibiotics (Table 2).

### Biofilm formation of *Acinetobacter* isolates

**Fig. 3:**
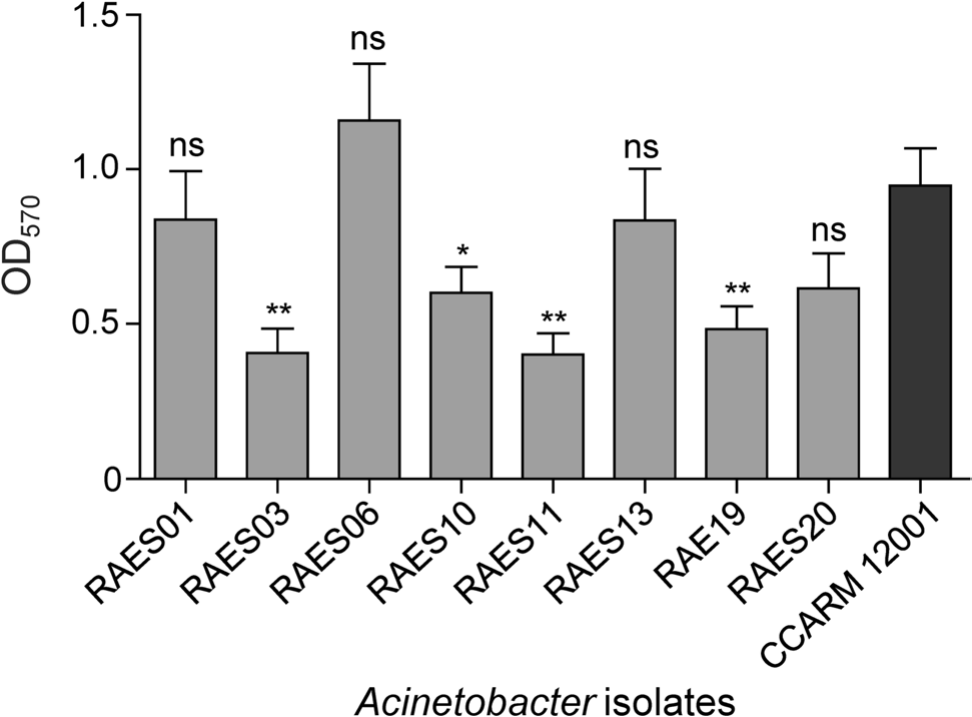
Biofilm formation of *Acinetobacter* isolates. Biofilm was quantified utilizing the crystal violet staining assay and measuring optical density at 570 (OD_570_). Isolates were incubated at 37°C for 24 h in static conditions. Experiments were repeated three times and the data represent mean ± standard deviation. Results were analyzed based on CCARM 12001 results by the Student’s *t*-test. *P < 0.05; **P < 0.01; ns, not significant

Previous study states that biofilm formation is one of the major virulence factors of *Acinetobacter.* (Dehbanipour and Ghalavand, 2022). To determine the extent of biofilm formation of each isolate, the biofilm formation assay was conducted and the biofilm formation ability of each isolate was compared to the MDR *A. baumannii* CCARM 12001 strain. RAES01, RAES06, RAES13, and RAES20 showed similar values with the control strain, exhibiting strong biofilm formation capability (Fig. 3). On the other hand, RAES03, RAES10, RAES11, and RAES19 showed low biofilm formation compared to the control strain (Fig. 3). Some reports claim the correlation between antibiotic resistance and biofilm formation in *Acinetobacter* (Babapour et al., 2016; Bardbari et al., 2017). However, our results do not show any correlation between antibiotic resistance and biofilm formation (Table 2 and Fig. 3). Considering that growth and motility affect biofilm formation of bacteria, further study is required for deeper analysis (Goller and Romeo, 2008). Our results support the idea that biofilm formation does not necessarily associate with antibiotic resistance in *Acinetobacter*.

### Motility assay of *Acinetobacter* isolates

**Fig. 4:**
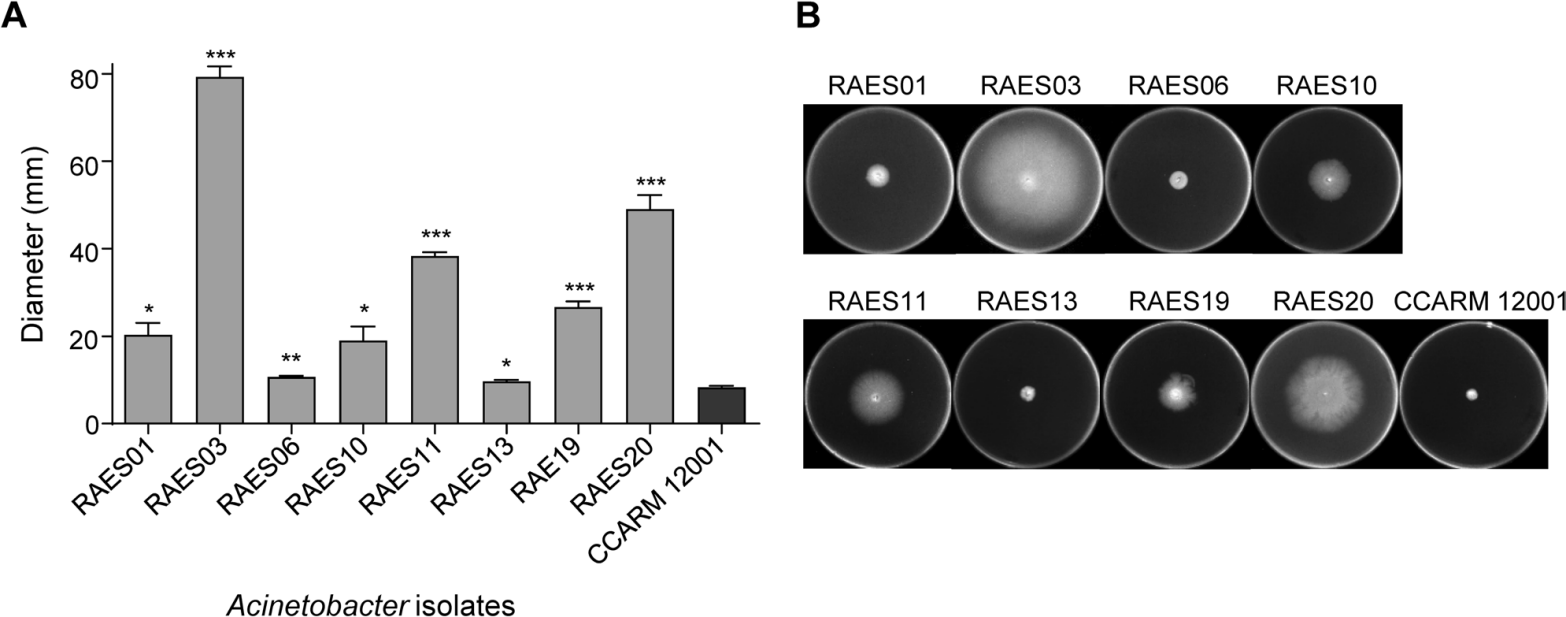
Swarming motility of *Acinetobacter* isolates. (**A**) Bar diagram of bacterial motility. The diameter of bacterial cells present on the plate was each measured for quantitative analysis. (**B**) Motility agar plates 24 h post-inoculation of *Acinetobacter* isolates. Experiments were repeated in triplicates and the data represent mean ± standard deviation. Results were analyzed based on CCARM 12001 results by the Student’s *t*-test. *P < 0.05; **P < 0.01; ***P < 0.001

Motility is one of the important virulence factors of *Acinetobacter* (Dehbanipour and Ghalavand, 2022). To compare the motility of the *Acinetobacter* isolates, we investigated the swarming motility with comparison to *A. baumannii* CCARM 12001. All isolates were highly motile compared to the control strain (Fig. 4). RAES06 showed the lowest motility among the isolates, which may be associated with its slow growth rate (Fig. 2). Interestingly, isolates with high biofilm-producing ability were revealed to have relatively low motility than low biofilm-forming isolates in our study (Figs. 3 and 4). Low motility is suggested to be coupled with higher adherence to surfaces, enhancing the efficiency of biofilm formation. Our results demonstrated the strong correlation between low motility and high biofilm formation in *Acinetobacter* spp. isolated from agricultural environments.

In conclusion, the findings in this study demonstrated that *Acinetobacter* isolates from agricultural fields in South Korea were resistant to many antibiotics and showed a high biofilm formation and motility capability. Since contaminated agricultural products can be a possible transmission route of MDR *Acinetobacter* spp., further investigation is needed to control and reduce MDR *Acinetobacter* spp. in agricultural environments.

### Supplementary Information

Below is the link to the electronic supplementary material.

Supplementary file1 (DOCX 26 KB)
