# Supplementary material for: Prevalence of antibiotic-resistant Acinetobacter spp. on soil and crops collected from agricultural fields in South Korea
Source: Food Sci Biotechnol. 2024 Jan 29;33(8):1931–7. doi: 10.1007/s10068-023-01496-7 (PMC11091005; doi:10.1007/s10068-023-01496-7)
Supplement: Supplementary file 1 — Supplementary file1 (DOCX 26 KB) [file 10068_2023_1496_MOESM1_ESM.docx]

**Table S1. Primers used in this study**

| **Primer** | **Sequence (5′-3′)** | **Reference** |
| --- | --- | --- |
| Acineto-specific-F | CTGCCTATTAGTGGGGGACA | (Fernando et al., 2016) |
| Acineto-specific-R | AAGGCACCAATCCATCTCTG |  |
| 27F | AGAGTTTGATCCTGGCTCAG | (Lane, 1991) |
| 1492R | GGTTACCTTGTTACGACTT |  |
|  |  |  |

**Table S2. Antibiotic resistance genes in *Acinetobacter* isolates**

| Strain | Colistin | | Chloramphenicol | | Streptomycin |
| --- | --- | --- | --- | --- | --- |
|  | *emrA* | *emrB* | *cat* | *craA* | *aadA* |
| RAES01 | + | + | + | + | + |
| RAES03 | + | + | + | + | - |
| RAES06 | + | + | + | + | + |
| RAES10 | + | + | + | + | + |
| RAES11 | + | + | + | + | + |
| RAES13 | + | + | + | + | + |
| RAES19 | + | + | + | + | + |
| RAES20 | + | + | + | + | + |
| Total (%) | 8/8 (100%) | 8/8 (100%) | 8/8 (100%) | 8/8 (100%) | 7/8 (87.5%) |

**References**

Fernando, D. M., Khan, I. U., Patidar, R., Lapen, D. R., Talbot, G., Topp, E., & Kumar, A. (2016). Isolation and Characterization of *Acinetobacter baumannii* Recovered from Campylobacter Selective Medium. *Front Microbiol, 7*, 1871. doi:10.3389/fmicb.2016.01871

Lane, D. (1991). 16S/23S rRNA sequencing. Nucleic acid techniques in bacterial systematics (Stackebrandt E & Goodfellow M, eds). In: Wiley, New York.
